# The Role of Decision-Making in Psychological Wellbeing and Risky Behaviours in Autistic Adolescents Without ADHD: Longitudinal Evidence from the UK Millennium Cohort Study

**DOI:** 10.1007/s10803-020-04783-y

**Published:** 2020-11-16

**Authors:** Mariko Hosozawa, William Mandy, Noriko Cable, Eirini Flouri

**Affiliations:** 1 Department of Epidemiology and Public Health, University College London, London, UK; 2 Department of Paediatrics and Adolescent Medicine, Juntendo University, Tokyo, Japan; 3 Research Department of Clinical, Educational and Health Psychology, University College London, London, UK; 4 Department of Psychology and Human Development, UCL Institute of Education, University College London, London, UK

**Keywords:** Autism spectrum disorder, Decision-making, Gambling task, Adolescence, Psychological wellbeing, Antisocial behaviours

## Abstract

This study examined the development of decision-making and its association with psychological wellbeing and risky behaviours in adolescents with and without autism. Participants included 270 autistic and 9,713 typically developing adolescents. In both samples, those with a diagnosis of attention-deficit/hyperactivity disorder (ADHD) were excluded. Data came from the Millennium Cohort Study, a nationally representative population-based birth cohort. Decision-making was assessed using the Cambridge Gambling Task at ages 11 and 14. Psychological wellbeing (happiness, self-esteem, depressive symptoms and self-harm) and risky/antisocial behaviours were self-reported at age 14. After adjusting for sex, cognitive ability, spatial working memory, socioeconomic status and pubertal status, autistic adolescents showed comparable quality of decision-making to that of their peers at both ages but also a more deliberative decision-making style as they aged. Only in autistic adolescents was this decision-making style associated with positive outcomes.

**Electronic supplementary material:**

The online version of this article (10.1007/s10803-020-04783-y) contains supplementary material, which is available to authorized users.

Autism spectrum disorder (ASD) is a neurodevelopmental condition characterized by difficulties in social interaction, communication and flexibility, as well as atypical sensory processing (American Psychiatric Association, 2013). Autistic children and adults, including those without intellectual impairment, also self-report difficulties in decision-making (Johnson, Yechiam, Murphy, Queller, & Stout, 2006; Luke, Clare, Ring, Redley, & Watson, 2012). These difficulties have, in turn, been suggested to at least partially explain the significant challenges experienced by many autistic people in their adulthood (e.g., low likelihood of independent living, high risk of unemployment, low self-determination and poor quality of life) (Chou, Wehmeyer, Palmer, & Lee, 2016; Robic et al., 2015; Shattuck et al., 2012). Such challenges may, in fact, start as early as during the transition from adolescence to adulthood, when social expectations of youth autonomous behaviour increase (Roux et al., 2013).

Therefore, understanding the development of decision-making in people with ASD and how it may relate to several areas of functioning, particularly during adolescence, could help to both improve our understanding of the condition and implement targeted interventions to support effective decision-making in this population. One way to measure decision-making skills is through gambling tasks that assess dynamic decision-making under risk. In typical development, such ‘objectively measured’ decision-making shows age-related, mostly linear, improvement from middle childhood to adolescence (Lensing & Elsner, 2018; Prencipe et al., 2011). However, there is only one cross-sectional study to date on how it develops across this period in people with ASD (Kouklari, Tsermentseli, & Monks, 2019). Thus, for people with ASD there is no evidence about within-person developmental change in decision-making under risk in childhood and, crucially, in adolescence, when dramatic physical, cognitive and social changes occur that may greatly influence decision-making (Blum, Astone, Decker, & Mouli, 2014). This period is also key for decision-making under risk due to the difference in the pace of maturation of the ventromedial prefrontal cortex compared to that of the dorsolateral prefrontal cortex. In contrast to the typically linear development of executive functions across childhood and adolescence, for example, affective decision-making abilities progress non-linearly, declining from late childhood to early adolescence and improving during mid-adolescence (Van Leijenhorst, Westenberg, & Crone, 2008). Adolescence is also a pivotal period in the life course when, in autistic people in particular, functional difficulties may increase, especially when compounded by comorbid mental health problems and challenging behaviours (Joshi et al., 2010).

Despite the lack of research on ‘naturally-occuring’ within-person change in ‘objectively measured’ decision-making in young people with ASD, there is much experimental research investigating differences in such decision-making between autistic and typically developing individuals in both childhood and adulthood. However, results are largely mixed for both children (Faja, Murias, Beauchaine, & Dawson, 2013; Johnson et al., 2006; Mussey, Travers, Klinger, & Klinger, 2015; South et al., 2014; Yechiam, Arshavsky, Shamay-Tsoory, Yaniv, & Aharon, 2010) and adults (South et al., 2008; Vella et al., 2018), with some studies reporting deficits and others reporting no differences (Vella et al., 2018). For example, a recent meta-analysis of studies using the Iowa Gambling Task (IGT), a widely used gambling task in studies of both typically developing individuals and those with neurodevelopmental conditions including ASD, showed that autistic people do not differ in IGT performance (Zeif & Yechiam, 2020).

These inconsistent results may be due to the studies’ very sample sizes (typically fewer than 50 autistic participants), the use of nonrepresentative samples introducing selection bias, the wide age-range of the participants (South et al., 2014; South et al., 2008; Yechiam et al., 2010), and the lack of controls for cognitive ability (such as IQ or executive function; Yechiam et al., 2010). In addition, for the studies using the IGT, where participants are required to learn to avoid the high-risk decks through trial and error (Brand, Recknor, Grabenhorst, & Bechara, 2007), performance may be influenced by contingency learning and/or flexibility rather than reflecting decision-making per se. This may be particularly problematic for autistic people who show repetitive and less flexible behaviours (De Martino, Harrison, Knafo, Bird, & Dolan, 2008; Johnson et al., 2006). Furthermore, while most experimental decision-making studies focused on the accuracy of response (i.e., quality of decision-making), there is an increased interest in measuring reaction time, a related but distinct cognitive process (Karalunas, Geurts, Konrad, Bender, & Nigg, 2014). Therefore, using tasks that are minimally sensitive to ‘learning’ – such as the Cambridge Gambling Task (CGT) – and assessing both accuracy and speed of decision-making may better capture the decision-making skills of autistic people (Deakin, Aitken, Robbins, & Sahakian, 2004). To date, only one study, with adults, has used the CGT in this population (Vella et al., 2018), and showed that quality of decision-making was similar to that of the comparison group but deliberation time was longer. The researchers hypothesized that a ‘deliberative decision-making style’ may be beneficial in some situations for autistic people but may also be exactly what underlies their difficulties in social contexts where rapid and intuitive reasoning is needed to make effective and efficient decisions. However, no study has tested that yet. We still do not know what the association is between decision-making style and important life outcomes among autistic people.

In summary, despite the importance of decision-making in daily life, very little is known about its development in the autistic population, particularly during adolescence, and how it may relate to life outcomes. In this study, we aimed to explore the similarities and differences in decision-making between those with and without autism during early and middle adolescence (ages 11 and 14) and to understand the role of, especially, accuracy and speed of decision-making in autistic and non-autistic adolescents’ psychological and behavioural outcomes (i.e., happiness, self-esteem, depressive symptoms, self-harm, antisocial behaviour, and risky health-related behaviours) at age 14. To overcome the methodological limitations of the extant literature, we drew our sample from a large population-representative birth cohort in the UK, therefore securing a relatively large number of autistic adolescents, and used the CGT. Due to lack of previous research we were not able to make specific predictions about either developmental change in the CGT or its links with psychological and behavioural outcomes in autistic adolescents. However, based on the one study to date using the CGT in autistic adults (Vella et al., 2018), we hypothesized that deliberation time would be longer in autistic adolescents. We also expected that it would be associated with less risky behaviour in them.

## Methods

### Sample

Our participants were drawn from the Millennium Cohort Study (MCS), a population-representative cohort study of 19,517 children born between September 2000 and January 2002 in the UK. To date information is available from data collected when the children were approximately 9 months and 3, 5, 7, 11 and 14 years. MCS used a stratified cluster design to oversample children living in disadvantaged areas and those, in England, with a high proportion of ethnic minority groups (details of the study design are described elsewhere; Connelly & Platt, 2014). MCS is approved by the UK National Health Service Research Ethics Committee, and written consent was obtained from all participating parents at each survey (Shepherd & Gilbert, 2019). The use of anonymized data for academic purposes did not require additional ethical approval.

At age 14, 11,872 children participated in MCS, and we used data from the 10,838 of them who completed the CGT. Out of this sample, our autism sample included children whose parent responded ‘yes’ to the question, used to ascertain ASD in other population-based studies (Baio et al., 2018): ‘Has a doctor or other health professional ever told you that your child had autism, Asperger’s syndrome or another autistic spectrum disorder?’ which was asked of the parents at each data wave from age 5 (i.e., at ages 5, 7, 11 and 14). The typically developing (TD) group consisted of children whose parent answered ‘no’ to that question. Children about whom there was not a valid response were excluded from the analysis (n = 5). Children with a history of accessing special education, or those with reports of learning disabilities, social or behavioural difficulties or neurological impairments (e.g., epilepsy) were excluded from the TD group (n = 855). Children who received a diagnosis of attention-deficit/hyperactivity disorder (ADHD) were excluded from both groups. Children who received an ASD diagnosis but then lost it (n = 37) were excluded from the autism group. Our final analytic sample included a total of 9,983 children (270 in the autism group, 9,713 in the TD group).

### Measures

#### The Cambridge Gambling Task (CGT)

The CGT is a measure of decision-making and risk-taking behaviour to obtain rewards (i.e., explicit risk contingencies), and was administered at the MCS children’s homes at ages 11 and 14. During the task, participants are asked to guess the location of a hidden token on a computer screen, and then gamble a proportion of their current points on their decision. Participants are presented with a row of ten boxes (red and blue) across the top of the screen and are told that the computer has hidden a “token” behind one of them. They have to choose a) which colour of box they believe the token is hidden behind (red or blue), and b) the number of points they want to gamble on their colour choice. The proportion of red to blue boxes (box ratio) varies during the task pseudo-randomly to assess the influence of statistical risk (probability) on decision-making. Participants start the task with 100 points and select a proportion of these points to bet on their decision (between 5% and 95%). A circle in the centre of the screen displays the current bet value, which either increases or decreases incrementally. Participants press the button when it shows the proportion of their score they would like to bet. These points are either added to, or subtracted from, their total score, depending on their decision and where the token was actually hidden. Before the formal experiment, participants are instructed with a practice phase to make sure they understand the rules. The task produces six outcome measures: (1) Risk-taking, the mean proportion of points bet on trials where the most probable colour was chosen. Higher scores reflect higher reward-sensitivity or lower punishment-sensitivity; (2) Quality of decision-making, the mean proportion of trials where the most probable colour was selected; (3) Deliberation-time, the mean time (in milliseconds) taken to make a box colour response; (4) Risk-adjustment, the extent to which betting behaviour is moderated by probability. It reflects the tendency to stake higher bets on high-probability compared to low-probability trials; (5) Delay-aversion, the time participants are prepared to wait in order to place a higher or lower bet; (6) Overall proportion bet, the mean proportion of points gambled across all trials. We excluded the overall proportion bet variable from the analysis as it was highly correlated (r > 0.9) with risk-taking. The remaining five measures are largely independent from each other and assess different aspects of decision-making tendency (Flouri, Moulton, & Ploubidis, 2019). Given the design of MCS, we accounted for age differences at the time the CGT was administered by converting the CGT scores to standardized z-scores adjusting for age in 6 months intervals for each age group (i.e., age 11 and age 14) using data from the whole MCS population.

#### Psychological Wellbeing

At age 14, children reported on the following aspects of psychological wellbeing: (a) happiness, measured by rating, on a 7-point Likert scale, their happiness in 6 life domains including school, appearance, family, friends, school work and life as a whole (α = 0.86, higher scores indicate more happiness; Patalay & Fitzsimons, 2016); (b) self-esteem, assessed with the Rosenberg self-esteem scale (Rosenberg, 1989; higher scores are indicative of higher self-esteem); (c) depressive symptoms, measured using the Short Mood and Feelings Questionnaire (SMFQ), a well-validated, for children and adolescents, self-report questionnaire of depressive symptoms in the past 2 weeks (range 0–26, higher scores indicate more depressive symptoms; Thapar & McGuffin, 1998), and (d) self-harming behaviour (self-reported self-harm in the past year).

#### Risky Behaviours

These were antisocial behaviour and engagement in risky health-related behaviours at age 14. Antisocial behaviour was measured by self-reported engagement in 15 behaviours (e.g., stealing, being rude in public spaces, damaging public property, carrying a knife or other weapon, hitting others, hurting others with a weapon, being questioned/warned/arrested by police, being a member of a street gang, engaging in internet hacking or launching cyber attacks by spreading malicious software, α = 0.69). Risky health-related behaviours were measured by self-reports about having ever drunk alcohol, smoked cigarettes, and used cannabis or other types of illegal drugs.

#### Spatial Working Memory

Studies in typical development have reported an association between executive function and overall quality of decision-making and deliberation time (Deakin et al., 2004). Therefore, we adjusted for spatial working memory, the only aspect of executive function available in MCS at the time of measurement of the CGT, in our model. This was measured with the CANTAB computer-based spatial working memory task at age 11. Participants were instructed to search for a token hidden under one of the boxes shown on the screen. The token never appeared in the same box twice and participants were advised not to return to a box that had already yielded a token. Performance was scored after a demonstration by the interviewer followed by three practice trials. An error was recorded if the participant searched the same box twice in a trial, and we used the total error score (the number of times a box was incorrectly searched) to control for spatial working memory in our study. We converted scores into standardized z-scores adjusting for age in 6 months intervals using data from the whole MCS population, and then reverse-coded them so that higher z-scores reflected better performance.

#### Covariates

We included the following variables as potential confounding factors, all of which were measured at age 11 unless otherwise indicated: sex, multiple birth indicator, general cognitive ability, highest parental education (at least ‘Advanced’ level, a qualification required to enter university), family poverty (household equivalized income less than 60% of the UK national median household income) and pubertal status at age 11 and 14 (at least some signs of puberty vs. no signs, measured according to the parent’s report for age 11 and the child’s report for age 14 as follows: signs of breast growth or menstruation or hair on body for females, and voice change or facial hair or hair on body for males). General cognitive ability was the mean age-adjusted score on three subscales of the British Ability Scales (BAS) II administered at the age 5 wave (the only MCS wave to date at which several non-verbal aspects of cognitive ability were available). These three measures were the BAS II Naming Vocabulary Subscale, measuring expressive language, the BAS II Picture Similarities Subscale, measuring problem-solving, and the BAS II Pattern Construction Subscale, measuring spatial ability.

### Statistical Analysis

#### Group Differences in CGT Z-Scores

We first derived descriptive statistics to summarize the data. Then, we examined the cross-sectional association between groups and the five CGT variables at age 11 and 14 using linear regression. To investigate age-related change in the CGT, we examined age 14 CGT z-scores controlling for age 11 CGT z-scores in linear regression models.

#### Association with Psychological Wellbeing and Risky Behaviours

To assess how CGT performance was associated with psychological wellbeing and risky behaviours at age 14, we examined (1) the cross-sectional association between age 14 CGT z-scores and the outcomes and (2) the longitudinal association between the change (between ages 11 and 14) in CGT z-scores and the outcomes. In view of our study aim to examine the specific roles of accuracy and speed of decision-making for these outcomes, we focused on the CGT variables of quality of decision-making and deliberation time. We used linear and logistic regression models and, as explained, we captured change by adjusting for age 11 CGT z-scores in the longitudinal analysis. To explore if associations differed by group, we included an interaction term for group*age 14 CGT score.

In all analyses, we first examined a crude model (Model 1), followed by a model adjusting for potential confounders (Model 2). Next, we adjusted for spatial working memory in Model 3. Furthermore, we conducted several sensitivity analyses, including further controlling for internalising and externalising symptom scores, measured using the parent-rated Strengths and Difficulties Questionnaire (Goodman, Lamping, & Ploubidis, 2010) at age 11, to test the robustness of the observed associations to emotional and behavioural problems which could confound the association between CGT and both psychological wellbeing and risky behaviours. All analyses were weighted using weights supplied by the MCS to account for the stratified sample, oversampling of subgroups and attrition. All analyses were conducted using Stata version 15 (Stata Corp, College Station, TX).

#### Missing Data

The proportion of data that were missing varied by variable, ranging from 0.01% (illegal drug use) to 9.3% (age 11 CGT). Missing data were imputed for covariates (imputed CGT at age 11 was included when used as a covariate in the ‘change’ models) using multiple imputation by chained equations (White, Royston, & Wood, 2011). The imputed results were broadly similar to those obtained using complete cases (Table S1).

## Results

Descriptive characteristics of the study sample are shown in Table 1. The children with autism, compared to the children in the TD group, were more likely to be male and from a low-income family and, on average, had lower cognitive ability and poorer spatial working memory. At age 14, children in the autistic group reported lower happiness, more depressive symptoms and more antisocial behaviour. The mean raw CGT scores for each group are shown in Table 2.

**Table 1:** Descriptive characteristics for each group (N = 9983)

	Typically developing (n = 9713)		Autism (n = 270)		P-value^a^
	n	%	n	%	
Descriptive characteristics					
Sex					< 0.001
Male	4651	49.5	202	77.8	
Female	5062	50.5	68	22.2	
Parental education					0.82
A-level^b^ or above	5423	50.6	152	49.7	
Below A-level^b^	3795	49.4	111	50.3	
Low household income ^c^					0.03
No	7247	75.0	188	67.1	
Yes	1995	25.0	75	32.9	
Signs of puberty at age 11					0.63
No	5805	65.2	175	66.9	
Yes	3101	34.9	86	33.1	
Signs of puberty at age 14					
No	745	7.5	30	11.1	0.07
Yes	8810	92.5	210	88.9	
Mean cognitive ability, SE	9084	53.9 (0.2)	240	50.2 (0.6)	< 0.001
Mean spatial working memory, z scores, SE	8853	0.1 (0.0)	246	− 0.3 (0.1)	< 0.001
Psychological wellbeing outcomes at age 14					
Mean happiness score, SE	9556	32.8 (0.1)	240	29.7 (0.6)	< 0.001
Mean self-esteem score, SE	9480	10.5 (0.1)	240	10.3 (0.3)	0.53
Mean depressive symptoms, SE	9517	5.6 (0.1)	237	7.4 (0.5)	< 0.001
Self-harming behaviour					
No	8218	84.8	196	80.4	0.16
Yes	1370	15.2	48	19.6	
Risky behaviours at age 14					
Mean antisocial behaviour score, SE	9411	0.9 (0.0)	233	1.2 (0.1)	0.01
Have drunk alcohol					0.01
No	5371	51.1	156	60.9	
Yes	4244	49.0	90	39.1	
Have smoked cigarettes					0.65
No	8232	83.5	207	82.0	
Yes	1357	16.5	38	18.0	
Have used illegal drugs					0.79
No	9203	94.6	237	95.1	
Yes	414	5.4	10	4.9	

Unweighted counts and weighted means and percentages are shown. N varies due to missing data. *SE* standard error

^a^P values from chi-square tests are shown for categorical variables and those from Wald tests are shown for continuous variables

^b^A-level is a qualification required to enter university

^c^Below 60% of UK national median household income

**Table 2:** Mean CGT raw scores for each group at baseline and follow-up

	Typically developing			Autism		
	n	Mean	SE	n	Mean	SE
Age 11						
Quality of decision-making	8818	0.81	0.00	242	0.78	0.02
Deliberation time (ms)	8818	3306.02	20.70	242	3493.59	111.59
Risk taking	8817	0.53	0.00	242	0.58	0.02
Risk adjustment	8817	0.69	0.02	242	0.47	0.10
Delay aversion	8794	0.29	0.00	236	0.30	0.03
Age 14						
Quality of decision-making	9713	0.88	0.00	270	0.85	0.01
Deliberation time (ms)	9713	2325.17	16.06	270	2663.57	83.41
Risk taking	9713	0.52	0.00	270	0.55	0.01
Risk adjustment	9713	0.99	0.02	270	0.88	0.07
Delay aversion	9710	0.27	0.00	269	0.30	0.02

Unweighted numbers and weighted means are shown

### Group Differences in CGT Z-Scores

The association between group (TD or ASD) and CGT z-scores was examined in the multivariable linear regression models shown in Table 3. In the unadjusted model, ASD diagnosis was associated with several CGT z-scores including lower quality of decision-making at age 14, longer deliberation time at age 14, greater risk-taking at both age 11 and 14, and lower risk adjustment at age 11. However, after adjusting for confounders in Model 2, only longer deliberation time at age 14 was associated with ASD diagnosis. This association was not explained by further adjusting for spatial working memory in Model 3. When we examined group differences in age-related change in CGT z-scores, the ASD group showed less reduction in deliberation time with age, which remained significant after adjusting for confounders and spatial working memory (Table 3). A sensitivity analysis substituting the general cognitive ability measure at age 5 for the cognitive ability measures available in the later waves (i.e., BAS word reading and BAS pattern construction at age 7 and BAS verbal similarities at age 11) did not change the overall pattern of findings on the CGT (results available on request).

**Table 3:** The effect of group status (TD or autism) on CGT z-scores at age 11 and 14 (TD as reference)

Outcome	Model 1 (unadjusted)		Model 2 (confounder adjusted)^a^		Model 3 (Model 2 + SWM adjusted)^b^	
	b	SE	b	SE	b	SE
Quality of decision-making						
Age 11	− 0.19	0.10	− 0.12	0.11	− 0.09	0.11
Age 14	− 0.22*	0.10	− 0.15	0.10	− 0.14	0.11
Changes between age 11 and 14^c^	− 0.16	0.10	− 0.11	0.10	− 0.14	0.11
Deliberation time						
Age 11	0.14	0.08	0.13	0.08	0.13	0.08
Age 14	0.36****	0.09	0.29****	0.09	0.24**	0.09
Changes between age 11 and 14^c^	0.32****	0.09	0.25****	0.09	0.18*	0.09
Risk taking						
Age 11	0.27**	0.10	0.09	0.09	0.07	0.09
Age 14	0.19*	0.08	0.00	0.08	-0.01	0.09
Changes between age 11 and 14^c^	0.10	0.08	− 0.01	0.08	− 0.07	0.08
Risk adjustment						
Age 11	− 0.22*	0.09	− 0.15	0.09	− 0.11	0.09
Age 14	− 0.12	0.08	− 0.09	0.08	− 0.07	0.10
Changes between age 11 and 14^c^	− 0.07	0.07	− 0.06	0.07	− 0.05	0.10
Delay aversion						
Age 11	− 0.02	0.11	− 0.03	0.11	− 0.04	0.11
Age 14	0.12	0.08	0.06	0.08	0.04	0.08
Changes between age 11 and 14^c^	0.11	0.09	0.06	0.08	0.05	0.08

*TD* typically developing, *SWM* spatial working memory, *SE* standard error

* *p* < 0.05; **p < 0.01; ***p < 0.005; ****p < 0.001

^a^Adjusted for sex, multiple birth indicator, parental education, household income, cognitive ability and pubertal status

^b^Further adjusted for spatial working memory

^c^CGT z-scores at age 14 controlling for age 11

### Association with Psychological Wellbeing and Risky Behaviours

The associations between CGT at age 14, changes in CGT between age 11 and 14, and psychological wellbeing and risky behaviours are shown in Tables 4 and 5. In general, quality of decision-making at age 14 was associated positively with psychological wellbeing (i.e., greater happiness, higher self-esteem, fewer depressive symptoms, less self-harming behaviour) and negatively with risky behaviours (i.e., less antisocial behaviour and less health-related risky behaviour) but the associations did not differ by group. Improvement in quality of decision-making during ages 11 and 14 was also associated positively with psychological wellbeing (i.e., greater happiness, fewer depressive symptoms, less self-harming behaviour) and negatively with antisocial behaviour and drug use (Table 4).

**Table 4:** The fully adjusted association between quality of decision-making (DM) z-scores and psychological wellbeing and risky behaviours at age 14, by group

Outcome at age 14	Quality of DM at age 14^a^		Changes in quality of DM^b^	
	b	SE	b	SE
Happiness (n = 9796)				
Quality of DM	0.36****	0.09	0.30***	0.09
Group	− 3.44****	0.56	− 3.43****	0.56
Group* Quality of DM	1.12	0.99	1.16	1.01
Self-esteem (n = 9720)				
Quality of DM	0.09*	0.04	0.07	0.04
Group	− 0.65**	0.24	− 0.65**	0.24
Group* Quality of DM	0.04	0.21	0.05	0.21
Depressive symptoms (n = 9754)				
Quality of DM	− 0.04**	0.01	− 0.04*	0.02
Group	0.47****	0.07	0.47****	0.07
Group* Quality of DM	− 0.01	0.07	− 0.01	0.07
Self-harm (n = 9832)				
Quality of DM	− 0.09*	0.04	− 0.11*	0.04
Group	0.78***	0.22	0.78***	0.22
Group* Quality of DM	0.18	0.18	0.19	0.17
Antisocial behaviour (n = 9644)				
Quality of DM	− 0.09****	0.02	− 0.07***	0.03
Group	0.22	0.15	0.22	0.15
Group* Quality of DM	0.16	0.15	0.15	0.15
Have drunk alcohol (n = 9861)				
Quality of DM	− 0.06*	0.03	− 0.04	0.03
Group^a^	− 0.31	0.17	− 0.31	0.17
Group* Quality of DM	0.24	0.14	0.23	0.14
Have smoked cigarettes (n = 9834)				
Quality of DM	− 0.12***	0.04	− 0.08	0.04
Group	0.05	0.22	0.05	0.22
Group* Quality of DM	0.02	0.23	− 0.00	0.24
Have used illegal drugs (n = 9864)				
Quality of DM	− 0.17**	0.06	− 0.14*	0.06
Group	− 0.14	0.41	− 0.14	0.41
Group* Quality of DM	0.61	0.38	0.61	0.40

*DM* decision-making, *SE* standard error. The typically developing group is taken as reference

* *p* < 0.05; **p < 0.01; ***p < 0.005; ****p < 0.001

^a^Adjusted for confounders (sex, multiple birth indicator, parental education, household income, cognitive ability, pubertal status and spatial working memory)

^b^ Adjusted for confounders + quality of DM z-score at age 11

**Table 5:** The fully adjusted association between deliberation time z-scores and psychological wellbeing and risky behaviours at age 14, by group

Outcome at age 14	Deliberation time at age 14^a^		Changes in deliberation time^b^	
	b	SE	b	SE
Happiness (n = 9796)				
Deliberation time	− 0.11	0.09	− 0.05	0.10
Group	− 3.94****	0.69	− 3.92****	0.68
Group* Deliberation time	1.15*	0.45	1.14*	0.45
Self-esteem (n = 9720)				
Deliberation time	− 0.06	0.04	− 0.05	0.04
Group	− 0.79***	0.26	− 0.78***	0.26
Group* Deliberation time	0.47*	0.22	0.47*	0.22
Depressive symptoms (n = 9754)				
Deliberation time	0.03*	0.01	0.03	0.02
Group	0.49****	0.07	0.49****	0.07
Group* Deliberation time	− 0.05	0.06	− 0.05	0.06
Self-harming behaviour (n = 9832)				
Deliberation time	0.08	0.04	0.08	0.05
Group c	0.74***	0.22	0.74***	0.22
Group* Deliberation time	− 0.02	0.18	− 0.02	0.18
Antisocial behaviour (n = 9644)				
Deliberation time	0.00	0.02	0.01	0.02
Group c	0.29	0.15	0.29	0.15
Group* Deliberation time	− 0.29***	0.10	− 0.29***	0.10
Have drunk alcohol (n = 9861)				
Deliberation time	0.02	0.03	0.00	0.03
Groupa	− 0.29	0.16	− 0.30	0.16
Group* Deliberation time	− 0.19	0.16	− 0.18	0.15
Have smoked cigarettes (n = 9834)				
Deliberation time	− 0.02	0.04	− 0.03	0.04
Group	0.13	0.23	0.13	0.23
Group* Deliberation time	− 0.33	0.26	− 0.33	0.26
Have used illegal drugs (n = 9864)				
Deliberation time	− 0.02	0.06	− 0.01	0.07
Group	− 0.15	0.39	− 0.15	0.39
Group* Deliberation time	− 0.12	0.51	− 0.13	0.51

*SE* standard error. The typically developing group is taken as reference

* *p* < 0.05; **p < 0.01;***p < 0.005; ****p < 0.001

^a^Adjusted for confounders (sex, multiple birth indicator, parental education, household income, cognitive ability, pubertal status and spatial working memory)

^b^Adjusted for confounders + deliberation time z-score at age 11

For the ASD group only, greater deliberation time at age 14 was associated with more happiness, higher self-esteem and fewer antisocial behaviours. Change in deliberation time between age 11 and 14 was also associated with more happiness, higher self-esteem and fewer antisocial behaviours in the ASD group only (Table 5). A sensitivity analysis further controlling for internalising and externalising symptoms at age 11 showed mainly similar results (Tables S2 and S3). We carried out another sensitivity analysis to explore group differences both in the CGT and in the association between the CGT and psychological wellbeing and risky behaviours when ADHD was not excluded throughout (Tables S4–S6). In that sample, the ASD group showed also less risk adjustment (at both ages), more delay aversion at age 14, and lower quality of decision-making at age 14. However, the associations between psychological wellbeing/risky behaviours and quality of decision-making or deliberation time were generally the same as those shown in the analysis of the samples excluding ADHD throughout.

## Discussion

Using data from a large general-population birth cohort which administered the CGT, a gambling task measuring decision-making under risk, at ages 11 and 14 years, we investigated the similarities and differences in the development of decision-making in autistic and non-autistic adolescents. We also explored how the speed and accuracy of decision-making (measured by the CGT variables of deliberation time and quality of decision-making, respectively) and their development across this period are related to real-life experiences (i.e., psychological wellbeing and risky behaviours) among autistic and non-autistic adolescents. After adjusting for possible confounders, autistic and TD adolescents showed comparable quality of decision-making at both age 11 and 14. However, autistic adolescents showed proportionately less reduction in deliberation time as they developed, compared to their TD peers, resulting in longer deliberation time at age 14. These were the only significant differences in decision-making skills, across the CGT domains, between the two groups. Furthermore, while quality of decision-making at age 14 and its improvement between age 11 and 14 were associated with better outcomes at age 14 in all adolescents, for autistic adolescents longer deliberation time at age 14, even after adjustment for deliberation time at age 11, was associated with better psychological wellbeing and fewer antisocial behaviours at age 14.

Our finding that quality of decision-making at both ages 11 and 14 was comparable between groups indicates that autistic adolescents have the same ability as their TD peers to make good decisions, at least during an experimental decision-making task. Our result is in line with another study using the CGT in adults which found similar performance between autistic and comparison groups (Vella et al., 2018). However, our result contradicts findings from other studies, including from a recent cross-sectional study that showed inferior quality of decision-making throughout from age 7 to age 10 in children with autism (Kouklari et al., 2019). Although we cannot give a definitive explanation for these contrasting findings, the different tasks used to measure decision-making skills (i.e., IGT in the previous study, CGT in our study) might have had a role to play. For example, the IGT relies more on contingency learning and flexibility than the CGT (Brand et al., 2007), as explained. Currently, there is no consensus on which of these experimental tasks is more appropriate for measuring decision-making in autistic people. It is important to reflect on which provides the most realistic approximation of real-life decision making. In fact, the less ‘controlled’ IGT may have the advantage of greater ecological validity because real-life decision-making is often a very complex and highly context-specific process. Related to this, it is important to reflect generally on how well such highly controlled (i.e., structured) decision-making tasks really represent the ‘open-ended’ nature of real-life decision-making.

Despite performing as well as their TD peers in quality of decision-making, autistic adolescents in our study took longer than their TD peers to make decisions as they aged. This result is in line with the evidence for self-reported slowness in decision-making in autistic people (Luke et al., 2012) and with some findings from experimental studies (Brosnan, Chapman, & Ashwin, 2014; Vella et al., 2018). Importantly, the longer deliberation time observed in our autistic participants remained significant after adjusting for both cognitive ability and spatial working memory. This evidence for a deliberative processing style is in line with other findings showing consistent differences in processing speed among autistic people (Velikonja, Fett, & Velthorst, 2019). It is also related to results from studies using non-social tasks that show a conservative response style (i.e., larger speed-accuracy trade-offs) as a specific characteristic of autistic children and adults (Karalunas et al., 2018; Pirrone, Dickinson, Gomez, Stafford, & Milne, 2017). However, our study is unique in showing that this deliberative decision-making style emerged in autistic people in adolescence. This may be due to autistic and non-autistic people likely following different developmental trajectories of ‘hot’ and ‘cool’ cognitive skills across childhood and adolescence. ‘hot’ decision-making skills in typical development are known to improve later than higher-order ‘cool’ cognitive skills (Prencipe et al., 2011). Thus, the ‘atypical’ or deliberative decision-making style of autistic people may have been present from childhood but could only ‘emerge’ in adolescence because TD individuals show their expected age-related improvement in deliberation time during this period. Relatedly, a recent longitudinal functional magnetic resonance imaging study demonstrated that in autism the lack of significant developmental changes in functional connectivity between the default mode network and the central executive network emerges during adolescence (Lawrence, Hernandez, Bookheimer, & Dapretto, 2019). Taken together, these findings suggest that deliberative decision-making may reflect a core autistic neurocognitive feature, possibly emerging as a specific characteristic of autistic people during adolescence.

Importantly, we found that only in autistic adolescents was such a deliberative decision-making style related to greater happiness and higher self-esteem as well as fewer antisocial behaviours. According to the dual processing theory, a deliberative response is associated with inhibitory and rule-based behaviour (Evans, 2008), which may explain the negative association between deliberative decision-making style and antisocial behaviour. The underlying mechanism between deliberative decision-making style and psychological wellbeing in autism could be more complicated. It may be that when autistic adolescents use a more deliberative decision-making style (i.e., effectively, when they process information in as much detail as they feel is appropriate) they show high levels of agency, in turn related positively to wellbeing. Previous experimental studies have reported that autistic participants gather more information than others before making decisions (Brosnan et al., 2014), even when penalised for doing so (Vella et al., 2018). It may be that by using a deliberative decision-making style, autistic adolescents are able to fulfil their need to pay attention to detail, which in turn makes them feel satisfied and happier. We note that this may be seen to contradict previous studies (Haigh, Walsh, Mazefsky, Minshew, & Eack, 2018; Hedvall et al., 2013), which showed that slower processing speed in general was associated with negative outcomes. However those studies often relied on ‘objectively measured’ outcomes which may not reflect the views of autistic people about themselves. Combining laboratory-based tasks with self-reported functioning in everyday life may help to identify strategies to best promote the wellbeing of autistic people with greater precision.

Our findings provide evidence for ways to better support decision-making in autistic adolescents and to promote positive psychological and behavioural outcomes in them. In previous studies, parents of autistic youth report low decision-making capacity in their children (Cheak-Zamora, Maurer-Batjer, Malow, & Coleman, 2019), and autistic people self-report avoidance in making decisions (Luke et al., 2012). However, our results revealed that, at least in an experimental task, autistic adolescents have the capacity to make decisions comparable to those of their TD peers. Disseminating this finding to parents, educators and adolescents themselves may change the predominant view about a decision-making deficit in autism and may help promote opportunities for autistic adolescents to develop and practise decision-making. Because decision-making skills are fostered through opportunity (Wehmeyer & Abery, 2013), it is important to create enough, and appropriate, opportunities for them.

Perhaps the key finding of our study is that deliberative decision-making (which was, in turn, typical among autistic adolescents as we showed) was associated positively with psychological wellbeing and negatively with antisocial behaviours in autistic but not in TD adolescents. Allowing autistic adolescents enough time to make decisions may therefore promote their mental health and help them in their transition to adulthood. Recent studies report the effectiveness of interventions aiming to promote self-determination, including decision-making skills, in this population (Nadig, Flanagan, White, & Bhatnagar, 2018). Therefore, considering this unique feature of ‘autistic’ decision-making when implementing these interventions may be useful in this regard as well.

### Limitations

This study has many strengths including the large sample size of adolescents with autism drawn from MCS, a population-representative birth cohort. The longitudinal design of the study allowed us to investigate age-related changes in decision-making during adolescence and how these may relate to various important outcomes in both TD and autistic adolescents. The rich information available in MCS allowed us to control for several possible confounders as well as test for several outcomes that are important both for functioning in adolescence and for transitioning to adulthood.

However, our study also has several limitations. First, the diagnosis of autism was based on parent report and was not confirmed externally. However, parent-reported autism diagnosis, used in many previous population-based studies, has been proven reliable (Baio et al., 2018; Daniels et al., 2012). Second, information about symptom severity was not available in MCS and it would be important in the future to understand how the severity of autistic symptoms may influence the development of decision-making. Symptom severity therefore could have confounded our findings but we note that, to date, the role of severity of autistic symptoms in decision-making has been inconsistent (Brosnan, Lewton, & Ashwin, 2016; Pirrone, Wen, Li, Baker, & Milne, 2018). Third, although we drew our sample from a population-based cohort and used weights and multiple imputation to account for sample attrition and item missingness, we were only able to include those children who took the CGT task at age 14. Fourth, we acknowledge that our conclusions are predicated on the assumption that taking longer to select a coloured box on the CGT, our gambling task, is evidence for increased “deliberation”. However, differences in perceptual processing times, motor response times, propensity for mind wandering, and the subjective value of getting out of the session are just some of the reasons why two people might differ in the time they take to execute a choice in this task. Fifth, nearly 90% of the autistic children in our sample had normal-range cognitive ability which is higher than would be expected (Baio et al., 2018). Therefore, the results of this study may be applicable only to those with normal-range cognitive ability. Nevertheless, even autistic people with normal-range cognitive ability self-report that they have difficulty in decision-making (Luke et al., 2012), making our study findings important. Sixth, our study population was autistic children without ADHD. As ADHD and ASD are highly comorbid, our findings cannot be generalised to the entire ASD spectrum. Seventh, the CGT is a standardized, ‘objective’ tool that measures decision-making with minimum sensitivity to learning ability, but is, as yet, untested in autistic children or adolescents.

In conclusion, this is the first longitudinal study to follow the development of decision-making in autism from early to middle adolescence. It found that autistic adolescents show comparable quality of decision-making to that of their TD peers at both early (age 11) and middle (age 14) adolescence, but also use a more deliberative decision-making style in middle adolescence. This decision-making style was independent of various confounders including cognitive ability and spatial working memory, suggesting that it may reflect a core autistic neurocognitive feature. Furthermore, only in the autism group was deliberative decision-making style associated with better psychological outcomes and less antisocial behaviour in middle adolescence. Considering how decision-making and its links with mental health and behaviour differ between autistic and TD adolescents may help to both support the decision-making of autistic adolescents and promote their wellbeing. We note however that most of our participants had normal-range cognitive ability, and none of them had ADHD, which may limit the generalizability of our findings across the ASD population.

## Electronic supplementary material

Below is the link to the electronic supplementary material.

Electronic supplementary material 1 (DOCX 45 kb)
