# Supplementary material for: The Role of Decision-Making in Psychological Wellbeing and Risky Behaviours in Autistic Adolescents Without ADHD: Longitudinal Evidence from the UK Millennium Cohort Study
Source: J Autism Dev Disord. 2020 Nov 16;51(9):3212–23. doi: 10.1007/s10803-020-04783-y (PMC8349343; doi:10.1007/s10803-020-04783-y)
Supplement: Supplementary file 1 — Electronic supplementary material 1 (DOCX 45 kb) [file 10803_2020_4783_MOESM1_ESM.docx]

Supplemental Table S1: Multiply imputed descriptive characteristics for each group (N = 9,983)

|  | Typically developing | Autism | p-value |
| --- | --- | --- | --- |
|  | % | % |  |
| Sex |  |  | < .001 |
| Male | 49.5 | 77.8 |  |
| Female | 50.5 | 22.2 |  |
| Low parental education |  |  | 0.97 |
| No | 49.7 | 49.5 |  |
| Yes | 50.3 | 50.5 |  |
| Low household income |  |  | 0.09 |
| No | 74.2 | 67.7 |  |
| Yes | 25.8 | 32.3 |  |
| Signs of puberty, age 11 |  |  | 0.72 |
| No | 65.2 | 66.5 |  |
| Yes | 34.8 | 33.5 |  |
| Signs of puberty, age 14 |  |  | 0.14 |
| No | 7.5 | 11.1 |  |
| Yes | 92.5 | 88.9 |  |
| Mean cognitive ability, SE | 53.7 (0.2) | 49.8 (0.6) | < .001 |
| Mean spatial working memory, SE | 0.06 (0.02) | -0.32 (0.08) | < .001 |

*Note:* Weighted percentages are shown.

Supplemental Table S2: The association between quality of decision-making (DM) z-scores and psychological wellbeing and risky behaviours at age 14 adjusted for internalising and externalising symptoms at age 11, by group

|  | Quality of DM at age 14^a^ | | Changes in quality of DM^b^ | |
| --- | --- | --- | --- | --- |
| Outcome at age 14 | b | SE | b | SE |
| Happiness (n = 9,796) |  |  |  |  |
| Quality of DM | 0.33^****^ | 0.09 | 0.28^***^ | 0.09 |
| Group | -1.67^**^ | 0.62 | -1.68^**^ | 0.62 |
| Group* Quality of DM | 1.24 | 1.02 | 1.27 | 1.03 |
| Self-esteem (n =9,720) |  |  |  |  |
| Quality of DM | 0.08 | 0.04 | 0.07 | 0.04 |
| Group | -0.01 | 0.26 | -0.01 | 0.26 |
| Group* Quality of DM | 0.09 | 0.20 | 0.09 | 0.20 |
| Depressive symptoms (n =9,754) |  |  |  |  |
| Quality of DM | -0.04^*^ | 0.02 | -0.04^*^ | 0.02 |
| Group | 0.21^***^ | 0.07 | 0.21^***^ | 0.07 |
| Group* Quality of DM | -0.03 | 0.08 | -0.04 | 0.08 |
| Self-harming behaviour (n =9,832) |  |  |  |  |
| Quality of DM | -0.08^*^ | 0.04 | -0.10^*^ | 0.04 |
| Group | 0.25 | 0.24 | 0.24 | 0.24 |
| Group* Quality of DM | 0.14 | 0.18 | 0.14 | 0.18 |
| Antisocial behaviour (n =9,644) |  |  |  |  |
| Quality of DM | -0.07^***^ | 0.02 | -0.06^*^ | 0.02 |
| Group | 0.09 | 0.15 | 0.09 | 0.15 |
| Group* Quality of DM | 0.15 | 0.14 | 0.14 | 0.15 |
| Have drunk alcohol (n = 9,861) |  |  |  |  |
| Quality of DM | -0.04 | 0.03 | -0.03 | 0.03 |
| Group^a^ | -0.35 | 0.18 | -0.35 | 0.18 |
| Group* Quality of DM | 0.25 | 0.14 | 0.24 | 0.14 |
| Have smoked cigarettes (n =9,834) |  |  |  |  |
| Quality of DM | -0.10^*^ | 0.04 | -0.06 | 0.04 |
| Group | -0.20 | 0.24 | -0.19 | 0.24 |
| Group* Quality of DM | -0.03 | 0.23 | -0.04 | 0.25 |
| Have used illegal drugs (n =9,864) |  |  |  |  |
| Quality of DM | -0.15^*^ | 0.06 | -0.12 | 0.06 |
| Group | -0.54 | 0.44 | -0.54 | 0.44 |
| Group* Quality of DM | 0.55 | 0.39 | 0.55 | 0.41 |

*Note*: DM = decision-making, SE = standard error. The typically developing group is taken as reference. ^*^*p* < .05, ^**^*p* < .01, ^***^*p* < .005, ^****^*p* < .001 ^a^ Adjusted for confounders (sex, multiple birth indicator, parental education, household income, cognitive ability, pubertal status, spatial working memory, and internalising and externalising symptoms at age 11). ^b^ Adjusted for confounders + quality of DM z-score at age 11

Supplemental Table S3: The association between deliberation time z-scores and psychological wellbeing and risky behaviours at age 14 adjusted for internalising and externalising symptoms at age 11, by group

|  | Deliberation time at age 14^a^ | | Changes in deliberation time^b^ | |
| --- | --- | --- | --- | --- |
| Outcome at age 14 | b | SE | b | SE |
| Happiness (n = 9,796) |  |  |  |  |
| Deliberation time | -0.05 | 0.09 | 0.00 | 0.10 |
| Group | -2.18^**^ | 0.77 | -2.17^**^ | 0.76 |
| Group* Deliberation time | 1.00^*^ | 0.49 | 0.99^*^ | 0.48 |
| Self-esteem (n =9,720) |  |  |  |  |
| Deliberation time | -0.04 | 0.04 | -0.04 | 0.04 |
| Group | -0.15 | 0.29 | -0.14 | 0.29 |
| Group* Deliberation time | 0.42 | 0.22 | 0.42 | 0.22 |
| Depressive symptoms (n =9,754) |  |  |  |  |
| Deliberation time | 0.02 | 0.02 | 0.02 | 0.02 |
| Group | 0.23^***^ | 0.08 | 0.23^***^ | 0.08 |
| Group* Deliberation time | -0.06 | 0.06 | -0.05 | 0.06 |
| Self-harming behaviour (n =9,832) |  |  |  |  |
| Deliberation time | 0.06 | 0.04 | 0.06 | 0.05 |
| Group ^c^ | 0.20 | 0.24 | 0.20 | 0.24 |
| Group* Deliberation time | 0.04 | 0.17 | 0.04 | 0.17 |
| Antisocial behaviour (n =9,644) |  |  |  |  |
| Deliberation time | -0.01 | 0.02 | 0.00 | 0.02 |
| Group ^c^ | 0.15 | 0.14 | 0.15 | 0.14 |
| Group* Deliberation time | -0.29^***^ | 0.10 | -0.29^***^ | 0.10 |
| Have drunk alcohol (n = 9,861) |  |  |  |  |
| Deliberation time | 0.01 | 0.03 | -0.01 | 0.03 |
| Group^a^ | -0.33 | 0.17 | -0.33 | 0.17 |
| Group* Deliberation time | -0.21 | 0.17 | -0.21 | 0.17 |
| Have smoked cigarettes (n =9,834) |  |  |  |  |
| Deliberation time | -0.05 | 0.04 | -0.05 | 0.04 |
| Group | -0.10 | 0.27 | -0.10 | 0.27 |
| Group* Deliberation time | -0.37 | 0.31 | -0.37 | 0.30 |
| Have used illegal drugs (n =9,864) |  |  |  |  |
| Deliberation time | -0.05 | 0.07 | -0.04 | 0.07 |
| Group | -0.55 | 0.41 | -0.55 | 0.41 |
| Group* Deliberation time | -0.12 | 0.61 | -0.13 | 0.61 |

*Note*: SE = standard error. The typically developing group is taken as reference. ^*^*p* < .05, ^**^*p* < .01, ^***^*p* < .005, ^****^*p* < .001 ^a^ Adjusted for confounders (sex, multiple birth indicator, parental education, household income, cognitive ability, pubertal status, spatial working memory, and internalising and externalising symptoms at age 11). ^b^ Adjusted for confounders + deliberation time z-score at age 11

Supplemental Table S4: The effect of group status (TD or autism) on CGT z-scores at age 11 and 14 (TD as reference) including children ever diagnosed with ADHD (N = 10,138)

|  | Model 1 (unadjusted) | | Model 2 (confounder adjusted)^a^ | | Model 3 (Model 2 + SWM adjusted)^b^ | |
| --- | --- | --- | --- | --- | --- | --- |
| Outcome | b | SE | b | SE | B | SE |
| Quality of decision-making |  |  |  |  |  |  |
| Age 11 | -0.28^**^ | 0.08 | -0.20^*^ | 0.09 | -0.16 | 0.09 |
| Age 14 | -0.32^***^ | 0.09 | -0.23^**^ | 0.09 | -0.20^*^ | 0.09 |
| Changes between age 11 and 14^c^ | -0.23^**^ | 0.08 | -0.17^*^ | 0.08 | -0.15 | 0.08 |
| Deliberation time |  |  |  |  |  |  |
| Age 11 | 0.15^*^ | 0.07 | 0.14 | 0.07 | 0.13 | 0.07 |
| Age 14 | 0.58^***^ | 0.16 | 0.50^**^ | 0.16 | 0.48^**^ | 0.16 |
| Changes between age 11 and 14^c^ | 0.53^**^ | 0.15 | 0.45^**^ | 0.15 | 0.43^**^ | 0.15 |
| Risk taking |  |  |  |  |  |  |
| Age 11 | 0.28^**^ | 0.08 | 0.07 | 0.08 | 0.05 | 0.08 |
| Age 14 | 0.20^**^ | 0.07 | -0.00 | 0.07 | -0.01 | 0.07 |
| Changes between age 11 and 14^c^ | 0.12 | 0.07 | -0.02 | 0.07 | -0.02 | 0.07 |
| Risk adjustment |  |  |  |  |  |  |
| Age 11 | -0.31^***^ | 0.08 | -0.22^**^ | 0.07 | -0.17^*^ | 0.07 |
| Age 14 | -0.25^***^ | 0.07 | -0.20^**^ | 0.07 | -0.16^*^ | 0.07 |
| Changes between age 11 and 14^c^ | -0.17^*^ | 0.07 | -0.15^*^ | 0.07 | -0.12 | 0.07 |
| Delay aversion |  |  |  |  |  |  |
| Age 11 | 0.15 | 0.09 | 0.07 | 0.09 | 0.06 | 0.09 |
| Age 14 | 0.25^**^ | 0.07 | 0.18^*^ | 0.07 | 0.16^*^ | 0.07 |
| Changes between age 11 and 14^c^ | 0.23^**^ | 0.08 | 0.17^*^ | 0.07 | 0.16^*^ | 0.07 |

*Note:* TD = typically developing; SWM = Spatial working memory; SE = standard error. ^*^*p* < .05, ^**^*p* < .01, ^***^*p* < .001 ^a^Adjusted for sex, multiple birth indicator, parental education, household income, cognitive ability and pubertal status. ^b^ Further adjusted for spatial working memory ^c^CGT z-scores at age 14 controlling for age 11

Supplemental Table S5: The association between quality of decision-making (DM) z-scores and psychological wellbeing and risky behaviours at age 14, by group including children ever diagnosed with ADHD (N = 10,138)

|  | Quality of DM at age 14^a^ | | Changes in quality of DM^b^ | |
| --- | --- | --- | --- | --- |
| Outcome at age 14 | b | SE | b | SE |
| Happiness (n = 9,922) |  |  |  |  |
| Quality of DM | 0.38^***^ | 0.09 | 0.32^***^ | 0.09 |
| Group | -3.13^***^ | 0.49 | -3.11^***^ | 0.50 |
| Group* Quality of DM | 0.58 | 0.72 | 0.59 | 0.73 |
| Self-esteem (n =9,845) |  |  |  |  |
| Quality of DM | 0.09^*^ | 0.04 | 0.08 | 0.04 |
| Group | -0.64^**^ | 0.22 | -0.64^**^ | 0.22 |
| Group* Quality of DM | -0.09 | 0.16 | -0.08 | 0.16 |
| Depressive symptoms (n = 9,880) |  |  |  |  |
| Quality of DM | -0.04^**^ | 0.01 | -0.04^*^ | 0.02 |
| Group | 0.53^***^ | 0.06 | 0.53^***^ | 0.06 |
| Group* Quality of DM | 0.03 | 0.06 | 0.03 | 0.06 |
| Self-harm (n =9,958) |  |  |  |  |
| Quality of DM | -0.10^*^ | 0.04 | -0.11^*^ | 0.04 |
| Group | 0.97^***^ | 0.18 | 0.98^***^ | 0.18 |
| Group* Quality of DM | 0.33^*^ | 0.15 | 0.33 | 0.15 |
| Antisocial behaviour (n = 9,765) |  |  |  |  |
| Quality of DM | -0.09^***^ | 0.02 | -0.08^**^ | 0.02 |
| Group | 0.26^*^ | 0.13 | 0.26 | 0.13 |
| Group* Quality of DM | 0.13 | 0.12 | 0.13 | 0.12 |
| Have drunk alcohol (n = 9,988) |  |  |  |  |
| Quality of DM | -0.06^*^ | 0.03 | -0.04 | 0.03 |
| Group^a^ | -0.24 | 0.14 | -0.24 | 0.14 |
| Group* Quality of DM | 0.20 | 0.11 | 0.20 | 0.11 |
| Have smoked cigarettes (n =9,959) |  |  |  |  |
| Quality of DM | -0.12^**^ | 0.04 | -0.08^*^ | 0.04 |
| Group | 0.13 | 0.18 | 0.12 | 0.18 |
| Group* Quality of DM | 0.00 | 0.16 | -0.01 | 0.17 |
| Have used illegal drugs (n =9,990) |  |  |  |  |
| Quality of DM | -0.17^**^ | 0.06 | -0.14^*^ | 0.06 |
| Group | 0.09 | 0.29 | 0.08 | 0.29 |
| Group* Quality of DM | 0.21 | 0.22 | 0.20 | 0.22 |

*Note*: DM=decision-making, SE = standard error. The typically developing group is taken as reference. ^*^*p* < .05, ^**^*p* < .01, ^***^*p* < .001 ^a^ Adjusted for confounders (sex, multiple birth indicator, parental education, household income, cognitive ability, pubertal status and spatial working memory). ^b^ Adjusted for confounders + quality of DM z-score at age 11

Supplemental Table S6: The association between deliberation time z-scores and psychological wellbeing and risky behaviours at age 14, by group including children ever diagnosed with ADHD (N = 10,138)

|  | Deliberation time at age 14^a^ | | Changes in deliberation time^b^ | |
| --- | --- | --- | --- | --- |
| Outcome at age 14 | b | SE | b | SE |
| Happiness (n = 9,922) |  |  |  |  |
| Deliberation time | -0.12 | 0.09 | -0.06 | 0.10 |
| Group | -3.60^***^ | 0.54 | -3.58^***^ | 0.54 |
| Group* Deliberation time | 0.75^***^ | 0.17 | 0.72^***^ | 0.17 |
| Self-esteem (n =9,845) |  |  |  |  |
| Deliberation time | -0.07 | 0.04 | -0.06 | 0.04 |
| Group | -0.73^**^ | 0.22 | -0.73^**^ | 0.22 |
| Group* Deliberation time | 0.27^***^ | 0.07 | 0.26^***^ | 0.07 |
| Depressive symptoms (n = 9,880) |  |  |  |  |
| Deliberation time | 0.03^*^ | 0.02 | 0.03 | 0.02 |
| Group | 0.53^***^ | 0.07 | 0.52^***^ | 0.07 |
| Group* Deliberation time | -0.02 | 0.02 | -0.02 | 0.03 |
| Self-harming behaviour (n =9,958) |  |  |  |  |
| Deliberation time | 0.08 | 0.04 | 0.08 | 0.05 |
| Group ^c^ | 0.90^***^ | 0.19 | 0.90^***^ | 0.19 |
| Group* Deliberation time | -0.01 | 0.08 | -0.01 | 0.08 |
| Antisocial behaviour (n = 9,765) |  |  |  |  |
| Deliberation time | 0.00 | 0.02 | 0.01 | 0.02 |
| Group ^c^ | 0.30^*^ | 0.13 | 0.30^*^ | 0.13 |
| Group* Deliberation time | -0.12^*^ | 0.05 | -0.13^*^ | 0.05 |
| Have drunk alcohol (n = 9,988) |  |  |  |  |
| Deliberation time | 0.01 | 0.03 | -0.01 | 0.03 |
| Group^a^ | -0.22 | 0.14 | -0.23 | 0.14 |
| Group* Deliberation time | -0.17 | 0.11 | -0.17 | 0.11 |
| Have smoked cigarettes (n =9,959) |  |  |  |  |
| Deliberation time | -0.02 | 0.04 | -0.03 | 0.04 |
| Group | 0.20 | 0.18 | 0.20 | 0.18 |
| Group* Deliberation time | -0.16 | 0.15 | -0.15 | 0.15 |
| Have used illegal drugs (n =9,990) |  |  |  |  |
| Deliberation time | -0.02 | 0.06 | -0.01 | 0.07 |
| Group | 0.09 | 0.28 | 0.09 | 0.28 |
| Group* Deliberation time | -0.12 | 0.22 | -0.13 | 0.21 |

*Note*: SE = standard error. The typically developing group is taken as reference. ^*^*p* < .05, ^**^*p* < .01, ^***^*p* < .001 ^a^ Adjusted for confounders (sex, multiple birth indicator, parental education, household income, cognitive ability, pubertal status, spatial working memory, and internalising and externalising symptoms at age 11). ^b^ Adjusted for confounders + deliberation time z-score at age 11
